# CircularSTAR3D: a stack-based RNA 3D structural alignment tool for circular matching

**DOI:** 10.1093/nar/gkad222

**Published:** 2023-03-29

**Authors:** Xiaoli Chen, Shaojie Zhang

**Affiliations:** Department of Computer Science, University of Central Florida, Orlando, FL 32816, USA; Department of Computer Science, University of Central Florida, Orlando, FL 32816, USA

## Abstract

The functions of non-coding RNAs usually depend on their 3D structures. Therefore, comparing RNA 3D structures is critical in analyzing their functions. We noticed an interesting phenomenon that two non-coding RNAs may share similar substructures when rotating their sequence order. To the best of our knowledge, no existing RNA 3D structural alignment tools can detect this type of matching. In this article, we defined the RNA 3D structure circular matching problem and developed a software tool named CircularSTAR3D to solve this problem. CircularSTAR3D first uses the conserved stacks (consecutive base pairs with similar 3D structures) in the input RNAs to identify the circular matched internal loops and multiloops. Then it performs a local extension iteratively to obtain the whole circular matched substructures. The computational experiments conducted on a non-redundant RNA structure dataset show that circular matching is ubiquitous. Furthermore, we demonstrated the utility of CircularSTAR3D by detecting the conserved substructures missed by regular alignment tools, including structural motifs and conserved structures between riboswitches and ribozymes from different classes. We anticipate CircularSTAR3D to be a valuable supplement to the existing RNA 3D structural analysis techniques.

## Graphical Abstract

**Graphical Abstract:**
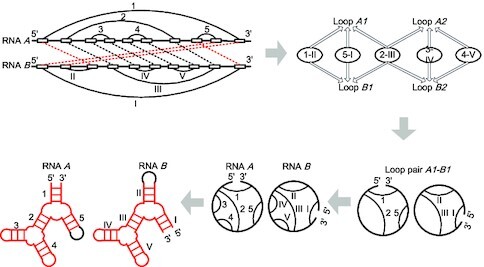
CircularSTAR3D: a stack-based RNA 3D structural alignment tool for circular matching.

## INTRODUCTION

The 3D structure of non-coding RNAs is one of the primary determinants of their functions. Comparing their structures can help researchers understand their functional and evolutionary relationships. A variety of RNA 3D alignment algorithms have been developed. One of the popular strategies is to represent RNA 3D structures by one-dimensional sequences consisting of structural alphabets (1–3), dihedral angles (4) or unit vectors (5,6). Then dynamic programming methods developed for sequence alignment are applied to the serialized data. The other group of methods takes into consideration the RNA base pairing interactions. For example, ARTS (7) identifies a conserved stack consisting of two base pairs, followed by a global extension process. STAR3D (8) and LocalSTAR3D (9) detect maximal sets of compatible conserved stacks and use them to guide the alignment of the remaining part of the RNA structures. SETTER splits RNAs into GSSUs (generalized secondary structure units) and uses the highly conserved units to guide the alignment (10). RMalign (11) and RNA-align (12) combine sequence and secondary structure alignments and calculate size-independent scores to choose the best alignments.

**Figure 1.**
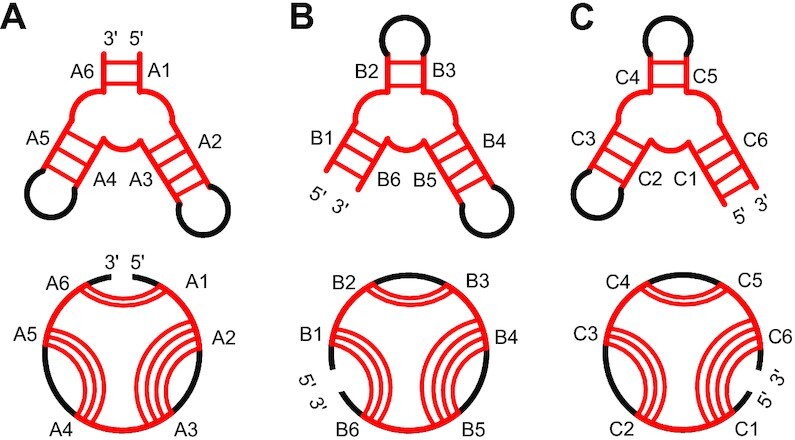
An example of circular matching. The aligned RNA structures are colored red. In each subfigure, the upper part is the secondary structure and the lower part is the circular diagram. In the circular diagram, each arc inside the circle represents a base pair.

Most of the existing RNA 3D structural alignment methods rely on sequence order. However, consistency of the sequence orders in the input RNAs is not required for structural similarity. As illustrated in Figure 1, the multiloops colored red in three subfigures can form the same 3D structure, while their sequence orders are different. In each subfigure, the strands in double-helix regions are labeled in their sequence order. For the RNA structure in Figure 1A, when reading from the 5′ end, the order of the strands is A1–A2–A3–A4–A5–A6. Taking the RNA structure in Figure 1A as the reference, the corresponding strands are B3–B4–B5–B6–B1–B2 in Figure 1B and C5–C6–C1–C2–C3–C4 in Figure 1C. It should be noted that the orders of the strands in Figure 1B and C are rotated. We define a pair of RNA 3D structures as a *circular match* if their 3D structures can be aligned when the sequence order is rotated in one of the RNAs. The terms circular match, circular alignment and rotated match are used interchangeably in this study. The circular matching problem for RNA structural motifs has been solved by aligning loop regions independently by using motif alignment tools such as RNAMotifScan and concatenating them in different ways (13–16). This method does not work for global or local RNA 3D structure alignment because most of the RNA motifs are hairpin loops, internal loops or multiloops, while larger RNA structures may contain many loops and stacks. The number of ways in which these can be selected and concatenated increases exponentially with the number of loops and stacks.

In recent years, circularly permutated RNA 3D structures, especially ribozymes, have drawn a lot of attention from researchers. For example, circularly permutated versions of group II introns were found in multiple bacterial phyla (17). A circularly permutated ribozyme scaffold was designed for engineering aptazymes and riboswitches (18). In addition to the circularly permutated RNA 3D structures, bioinformatics methods are employed to predict hairpin ribozymes in circular RNAs (19). Circularly comparing the RNA 3D structures can facilitate these studies. However, there is still a lack of computational tools that can find circular matches between RNA 3D structures.

In this study, we report an RNA 3D structural alignment tool for circular matching, named CircularSTAR3D, to handle the circular matching problem. For a pair of RNAs, CircularSTAR3D outputs all the circular matches between them. It is important to note that the alignment procedure only relies on the structural information of the input RNAs. CircularSTAR3D first searches rotated matched internal loops and multiloops that are closed by conserved stacks. It then iteratively extends the stack sets and aligns the loop regions under the guidance of the aligned stacks. CircularSTAR3D has been tested on a non-redundant dataset, showing that circular matching is a common phenomenon in RNA 3D structures. We further examined three applications of circular matching, namely structural motifs, riboswitches and ribozymes.

## MATERIALS AND METHODS

### Detect the conserved stack pairs

**Figure 2.**
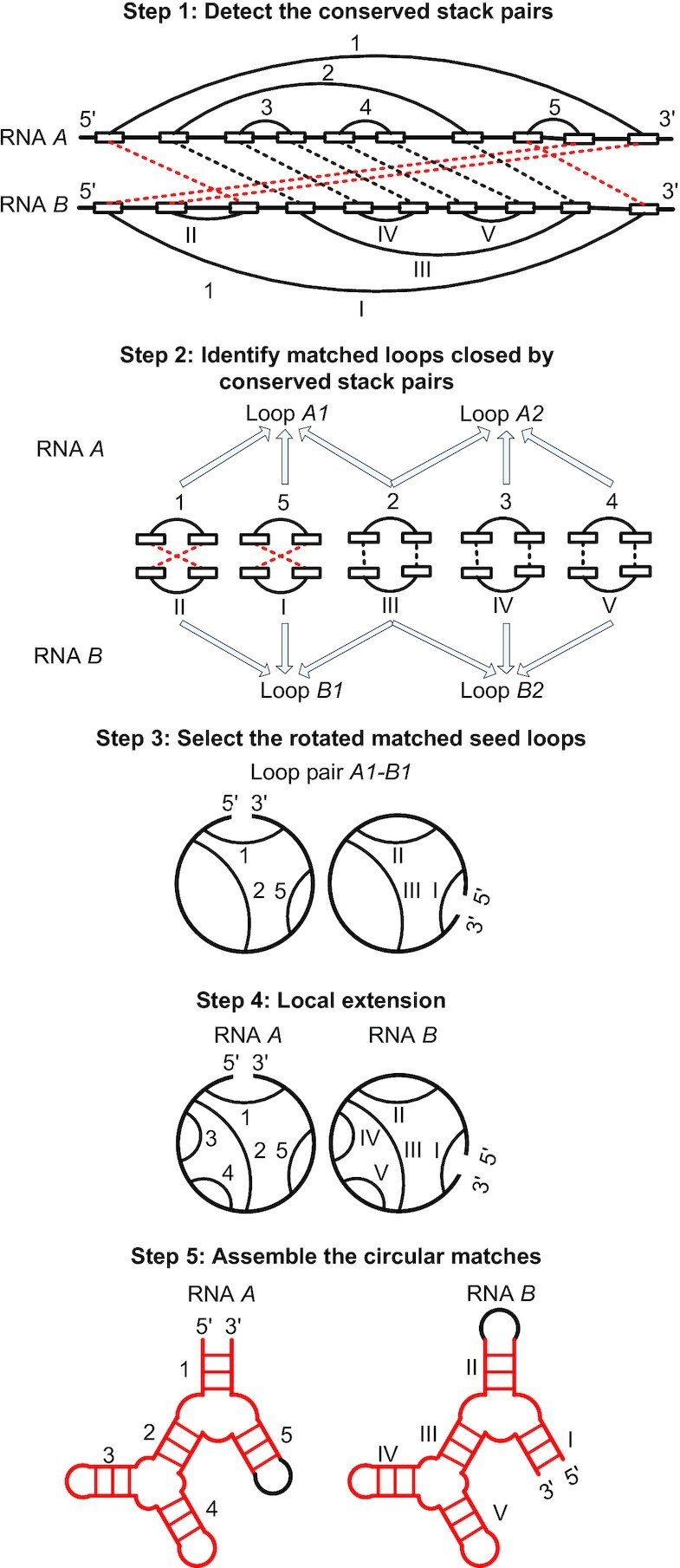
Key steps in CircularSTAR3D. In Step 1, the boxes linked by the arcs indicate the complementary strands in stacks. The dashed lines show the matched stack pairs between RNA *A* and RNA *B*. The rotated matched stack pairs are indicated by red dashed lines. In Step 2, the arrows indicate the stacks that close the corresponding loops. In Step 3, the secondary structures are represented by circular diagrams, where each arc in the diagram represents a stack. In Step 4, stack pairs 3–IV and 4–V are added into the alignment during local extension. In Step 5, the rotated matched RNA 3D structures between RNA *A* and RNA *B* are colored red.

CircularSTAR3D takes a pair of RNA 3D structures as input in PDB or PDBx/mmCIF format. CircularSTAR3D uses DSSR v1.5.3 (20) to annotate the base pairing interactions. From the base pairing annotation and the atomic coordinates, CircularSTAR3D detects the conserved stacks between the input RNAs. The terms conserved stack and conserved stack pair are used interchangeably in this study. A stack is formed by a set of consecutive nested base pairs. A conserved stack pair is a pair of stacks with root mean square deviation (RMSD) lower than a threshold (the default value is 4  Å). Similar to STAR3D and LocalSTAR3D, CircularSTAR3D uses the conserved stacks to guide the alignment. Different from STAR3D and LocalSTAR3D, CircularSTAR3D allows circular matching when detecting the conserved stacks. An example is shown in Step 1 of Figure 2. The circular matching in conserved stacks is indicated by red dashed lines. Stack 1 in RNA *A* and stack II in RNA *B* form a circular match. Stack 5 in RNA *A* and stack I in RNA *B* also form a circular match.

### Identify matched loops closed by conserved stacks pairs

For a pair of input RNAs, CircularSTAR3D may detect as many as a million conserved stacks due to partially overlapped stacks. STAR3D and LocalSTAR3D consider top-ranked conserved stacks with the longest length and the lowest RMSD. To achieve a short running time and high sensitivity for circular matched substructures, CircularSTAR3D considers all the stacks that close the internal and multiloops. Specifically, CircularSTAR3D first extracts the internal loops and multiloops from DSSR’s annotation for both input RNAs. Then it identifies the closing base pairs of each loop. The conserved stacks containing the closing base pairs are kept. Thus, a set of matched loops closed by conserved stacks are generated. In the example shown in Step 2 of Figure 2, loop *A1* is closed by stacks 1, 5 and 2, while loop *B1* is closed by stacks II, I and III. Conserved stack pairs 1–II, 5–I and 2–III are detected in Step 1. Therefore, loop *A1* and loop *B1* form a matched loop. For the same reason, loop *A2* and loop *B2* form a matched loop.

### Select the the rotated matched seed loops

CircularSTAR3D focuses on circular matches between the input RNAs. Therefore, from the matched loops identified in the last subsection, it only keeps the potential rotated matched loop pairs as seed loops. The potential rotated matched seed loops are the loop pairs that are closed by at least one rotated matched stack pair. As shown in Step 3 of Figure 2, loop pair *A1*–*B1* is selected as a rotated matched seed loop because it is closed by rotated matched stack pairs 1–II and 5–I. Loop pair *A2*–*B2* is discarded.

### Local extension

To find the consensus of conserved stacks, STAR3D and LocalSTAR3D construct compatible graphs by using juxtaposing and enclosing relationships, where each node represents a conserved stack. STAR3D calculates the maximum clique in the compatible graphs to generate a compatible stack pair set. To generate the local alignment, LocalSTAR3D adds adjacency constraints to the compatible graph and calculates the maximal subgraphs that are cliques with the compatible edges and connected components with the adjacency edges.

**Figure 3.**
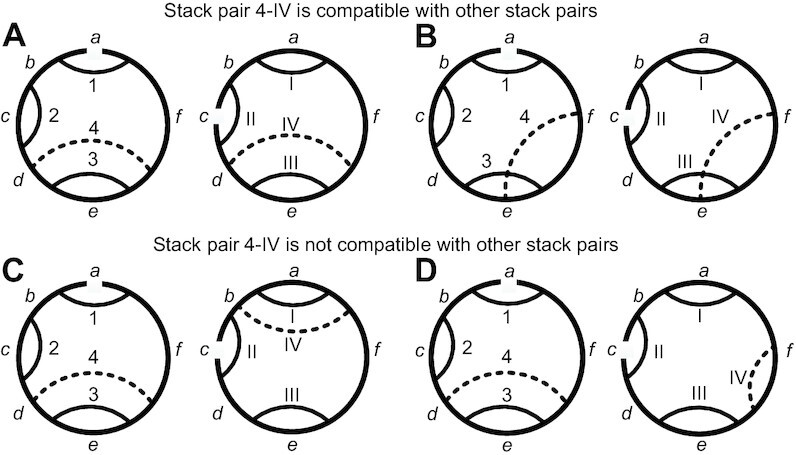
Examples of compatible and incompatible stack pairs. Each RNA has a counterclockwise 5′ to 3′ orientation. 1–I, 2–II and 3–III are the stack pairs selected in previous iterations. 4–IV is a candidate stack pair that needs to check the compatibility with the existing stack pairs.

Using the same idea, for each rotated matched seed loop, CircularSTAR3D calculates the maximal compatible stack pair sets. The initial conserved stack sets contain the conserved stacks closing the seed loops. Then new conserved stacks are added to the sets iteratively. Here we define a new type of topology relationship that allows circular matching. When adding a new conserved stack pair, it must be at the same interval as in the existing stack pairs. Figure 3 shows examples of compatible and incompatible stack pairs. The initial stack pair set includes the stack pairs 1–I, 2–II and 3–III. They divide both RNAs into intervals that are labeled with letters from *a* to *f*. The corresponding intervals in each RNA are labeled with the same letters. Now we evaluate the compatibility of the candidate conserved stack pair 4–IV with the initial stack pairs. For example, in Figure 3A, the strands of both stack 4 and IV are at intervals *d* and *f*. Therefore conserved stack pair 4–IV in Figure 3A is considered compatible with the initial conserved stack pairs. In Figure 3C, the strands of stack 4 are at intervals *d* and *f*, while those of stack IV are at intervals *b* and *f*. Therefore conserved stack pair 4–IV in Figure 3C is considered not to be compatible with the initial conserved stack pairs. Besides the compatibility requirement in topology, the RMSD for each set of compatible stack pairs must be lower than a threshold (the default value is 4  Å). This topology relationship takes into consideration the stack pairs that form pseudoknots. As shown in Figure 3B, CircularSTAR3D allows crossed stacks, which is not supported by STAR3D and LocalSTAR3D.

With the aforementioned topology relationship, CircularSTA3D uses a traversal strategy based on the Bron–Kerbosch algorithm to generate the maximal compatible stack pair sets (21). CircularSTAR3D iteratively searches for conserved stack pairs within a fixed distance from the outermost stack pairs. The default distance is 15 nucleotides, which is the same as the default value of LocalSTAR3D’s adjacent constraint. An example of one iteration is shown in Step 4 in Figure 2. In the local extension phase, CircularSTAR3D does not remove the initial stack pairs during traceback to make sure that the rotated matched seed loops are always in the alignment. A rotated matched seed loop may associate with multiple stack pair sets because two loops may have circular matches in different ways, as illustrated in Figure 1. In the development of CircularSTAR3D, an alternative topology relationship was evaluated, which considers the compatibility for each triplet of conserved stack pairs. However, we found that pre-computing the triplet relationship was more time-consuming and required different processing methods for internal loops and multiloops.

### Assemble the circular matches

STAR3D and LocalSTAR3D construct a tree-like consensus of conserved stack pairs. The mapping of the loop intervals in the input RNAs is obtained by traversing the tree-like consensus. CircularSTAR3D follows a similar process with two major differences. First, by using the new compatibility definition illustrated in Figure 3, CircularSTAR3D constructs a circular consensus. The conserved stack pairs divide the other regions in the RNA structures into circular ordered loop intervals. The mapping of the loop intervals can be obtained by traversing the circle. The second major difference is in the loop alignment. Modifications are made in CircularSTAR3D to allow circular matching for each loop pair. As the example shows in Step 4 in Figure 2, the hairpin loop closed by stack 5 in RNA *A* maps to the dangling ends linked to stack I in RNA *B*. Taking the sequence order in RNA *A* as the reference, CircularSTAR3D will align the 3′ end before the 5′ end in RNA *B*.

Similar to LocalSTAR3D (9), after aligning the stacks and the loop regions, CircularSTAR3D assembles them into candidate local alignments and filters the candidates with RMSD greater than a threshold (the default value is 4  Å). For each local alignment, CircularSTAR3D calculates an alignment score based on the number of aligned, deleted and inserted nucleotides. The local alignments in the output are first sorted by the alignment scores, and then by the RMSD values.

## RESULTS

### Alignment quality assessment with the R-FSCOR dataset

We use the R-FSCOR dataset to evaluate the alignment quality of CircularSTAR3D. The R-FSCOR dataset is a non-redundant RNA structure list that has been used as the benchmark dataset in previous RNA 3D structure alignment studies (6,8,9). In this study, the implementation of CircularSTAR3D was tested on a Linux desktop with an Intel Core i7-8700 CPU. We used CircularSTAR3D to generate the all-to-all alignments for the RNAs in the R-FSCOR dataset. The combination of the 192 RNAs in the R-FSCOR dataset generates 18 336 RNA pairs in total (we do not compare an RNA with itself). CircularSTAR3D output circular alignments for 2966 RNA pairs (16.2% of total RNA pairs). CircularSTAR3D did not output alignments when there is no conserved stack or no rotated matched loop detected in the input RNA pairs.

We noticed that local RNA structural alignment tools may generate fragmented alignments. In these cases, it is not suitable to evaluate the alignments using the total number of aligned nucleotides or base pairs. When evaluating our local alignment tool LocalSTAR3D (9), we defined Aligned Connected Structure (ACS), Percentage of Connected Structural Identity (PCSI) and Percentage of aligned Connected Secondary Structure (PCSS). An ACS is an aligned substructure where for each nucleotide, there is a nucleotide in this substructure which either is within a distance threshold from it (five nucleotides in our study) or forms a base pair with it. Based on the definition of ACS, PCSI is defined as the percentage of aligned nucleotides within 4  Å in the largest ACS with respect to the length of the shorter sequence, and PCSS is defined as the percentage of aligned base pairs within 4 Å in the largest ACS with respect to the smaller number of base pairs in the input RNAs. In this study, we followed the evaluation methods in LocalSTAR3D and used the same metrics (9).

To the best of our knowledge, there are no existing tools that can find the rotated matched RNA 3D structures. Therefore, we cannot assess CircularSTAR3D’s alignment quality by comparing it with other tools. CircularSTAR3D calculates the alignment by a ‘two-step’ strategy, identification of rotated matched seed loops and the local extension. Here we investigated the quality of each step’s results by using PCSI and PCSS. Similar to LocalSTAR3D, CircularSTAR3D was designed to report more than one aligned region between an input RNA pair. Therefore, we assessed the seed loops and the alignments after the local extension in the top two alignments for each input RNA pair.

**Figure 4.**
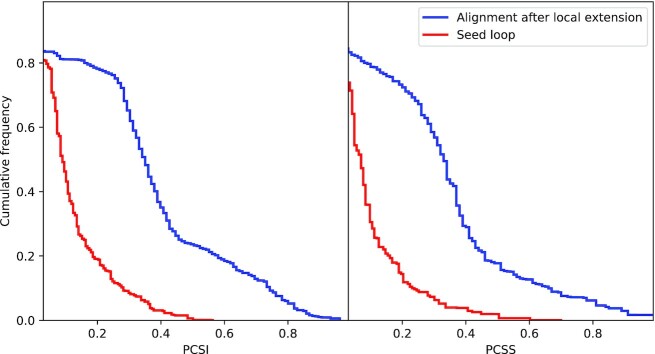
The cumulative frequencies of the PCSI and the PCSS values in CircularSTAR3D’s best alignments. The blue curves represent the PCSI and the PCSS values in the alignments after the local extension. The red curves represent the PCSI and the PCSS values in the seed loops.

The cumulative frequencies of the PCSI and PCSS for the best alignments are shown in Figure 4. In the best alignments, the average PCSI and PCSS values of the seed loops are 0.118 and 0.095 (red curves). The average PCSI and PCSS values of the alignments after the local extension are 0.363 and 0.325 (blue curves). The PCSI and PCSS values of the alignments after the local extension are about three times those of the seed loops. The cumulative frequencies of the PCSI and PCSS for the second best alignments are shown in Supplementary Figure S1. In the second best alignments, the average PCSI and PCSS values are 0.114 and 0.091 for the seed loops (red curves) and 0.348 and 0.306 for the alignments after local extension (blue curves). The differences in average PCSI and PCSS between the best alignments and the second best alignments are small (0.017 and 0.019), which suggests that the second best alignments are also potential candidates for circular match analysis.

### Structural alignments for motifs

Kink-turn motifs are well-known RNA internal loop motifs with sharp turns (kinks) (22). For example, in the *Haloarcula marismortui* 23S rRNA (PDB 1s72, chain 0), nine instances of kink-turn motifs have been identified (14,23). By using a kink-turn 3D structure (PDB 4bw0, chain A) (24) as the query, LocalSTAR3D found six out of the nine kink-turn motif instances (9). Using the same query, CircularSTAR3D found two additional kink-turn motif instances. CircularSTAR3D and LocalSTAR3D found eight out of nine kink-turn motif instances in total. The additional two instances found by CircularSTAR3D are shown in Supplementary Figure S2.

**Figure 5.**
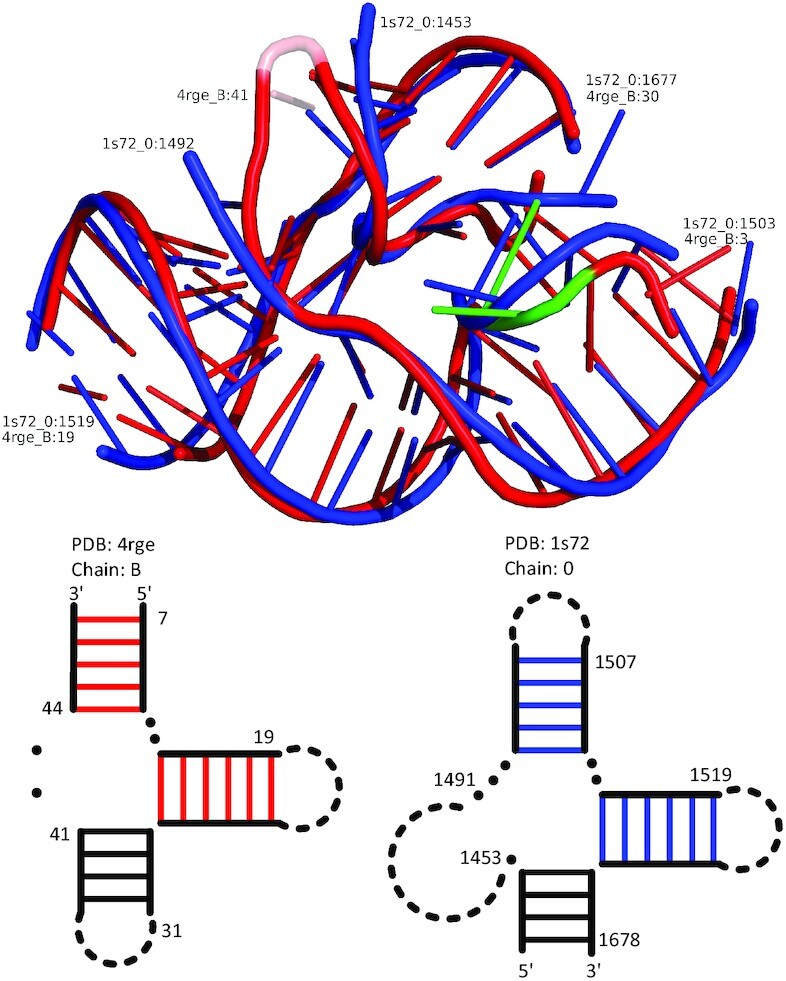
The circular matching instances for a multiloop motif in *H. marismortui* 23S rRNA (PDB 1s72, chain 0). The upper part of the figure shows the superimposition of the aligned regions, where the red tubes represent the aligned regions in the twister ribozyme and the blue tubes represent that in the rRNAs. The insertions and deletions in the alignment are shown in half-transparent tubes. The twister ribozyme’s self-cleavage sites are indicated in green. The lower part of the figure shows the secondary structures of the aligned regions, in which the twister ribozyme is on the left and the rRNA is on the right. In the secondary structures, the red and the blue lines indicate the base pairs in the conserved stacks that are used as anchors in the alignment process; the solid lines show the aligned regions; and the dashed lines are the rest of the regions for completeness.

In addition to the internal loop motifs, CircularSTAR3D can also detect circular matching instances for multiloop motifs. An example is shown in Figure 5. This multiloop motif was first discovered by aligning loop regions with RNAMotifScan and enumerating different ways to concatenate them (13,16). The motif instances can be found in an env22 twister ribozyme (PDB 4rge, chain B) (25) and rRNAs. Twister is a small self-cleaving ribozyme that can be found in many bacteria and eukaryota (26). The main component of the twister ribozyme is a three-way multiloop. Interestingly, this three-way multiloop can be rotated matched to a substructure present in rRNAs. Figure 5 shows the instance in *H. marismortui* 23S rRNA (PDB 1s72, chain 0). The instances in three other rRNAs are shown in Supplementary Figure S3. In the alignments of these motif instances, only two of the three conserved stacks are detected and used as anchors. However, CircularSTAR3D still includes the whole multiloop in the alignment, illustrating the robustness of our method. Compared with the alignments generated by RNAMotifScan (16), CircularSTAR3D aligns not only the multiloops but also the substructures around them. More importantly, CircularSTAR3D’s alignments contain the self-cleavage sites, U5 and A6 in the twister ribozyme, which are colored green in the 3D structures. The self-cleavage sites adopt a splayed-apart conformation that is important to cleavage catalysis and structural integrity. The corresponding sites in rRNAs share a similar splayed-apart conformation, suggesting potential functional relationships.

### Structural alignments for riboswitches

Riboswitches are metabolite-binding RNAs that are important in gene expression regulation (27). Distinct classes of riboswitches are established based on the sequence and structure conservation (28). In most riboswitch classes, the aptamer domains are more conserved than the gene control platform domains. This is because the aptamer domain must fold into a specific structure to bind to the metabolites. For example, the aptamer domain in SAM-II has a classical or H-type pseudoknot structure (27).

**Figure 6.**
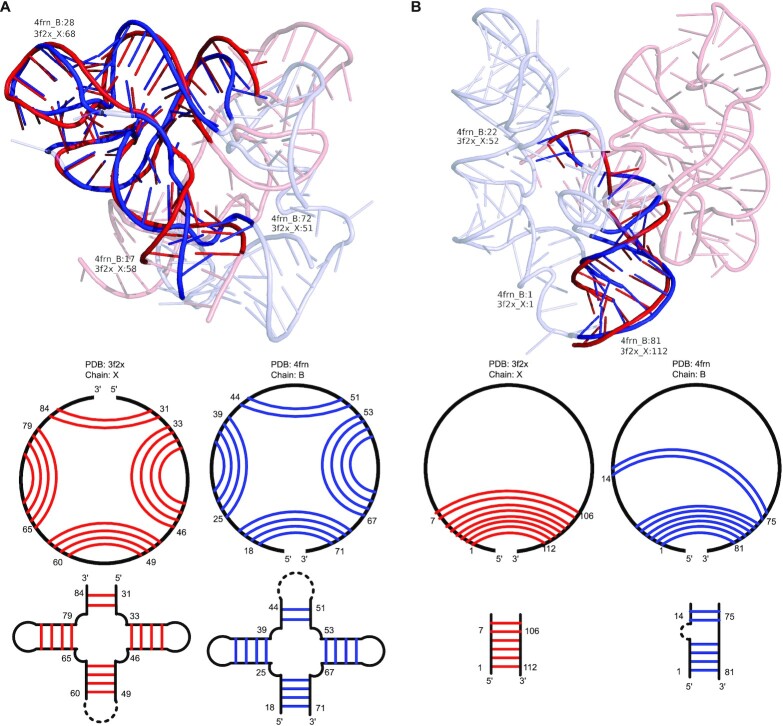
The structure alignment between a cobalamin riboswitch (PDB 4frn, chain B) and an FMN riboswitch (PDB 3f2x, chain X) generated by CircularSTAR3D and LocalSTAR3D. The upper part of each subfigure shows the superimposition of the aligned regions, where the red tubes represent the aligned regions in the FMN riboswitch and the blue tubes represent those in the cobalamin riboswitch. The regions outside the alignment are shown in half-transparent tubes. The lower part of each subfigure is the secondary structure of the aligned regions represented by using circular diagrams and cartoons. In the circular diagrams, the red curves and blue curves indicate the base pairs in the conserved stacks. In the cartoons, the red lines and blue lines indicate the base pairs in the conserved stacks. (**A**) The alignment generated by CircularSTAR3D. (**B**) The alignment generated by LocalSTAR3D.

We downloaded the riboswitch structures in the ‘Representative Sets of RNA 3D Structures’ list (version 3.141) on the BGSU RNA site by using ‘riboswitch’ as the search filter (29). The dataset contains 59 representative riboswitch structures. CircularSTAR3D was used to perform all-to-all alignment for the structures in this dataset. We found 68 alignments that contain circular matches with positive alignment scores and further obtained 50 alignments that have better coverage than the best alignments generated by LocalSTAR3D (see Supplementary Table S1). It should be noted that all these alignments are between different riboswitch classes. An example is shown in Figure 6, where the blue tube is a cobalamin riboswitch (PDB 4frn, chain B) (30) and the red tube is an FMN riboswitch (PDB 3f2x, chain X) (31). Both RNA structures are in the ligand-binding status. Although these two riboswitches bind different metabolites and only share 56.8% sequence identity, the 3D structures of their aptamer domains are surprisingly similar to each other. Figure 6A shows the CircularSTAR3D’s alignment for these two riboswitches. The alignment contains 55 nucleotides and has an RMSD of 3.21  Å. This similarity may suggest common features in the aptamer domain across different riboswitch classes. Interestingly, the similarity can only be detected by circular matching. The best alignment generated by LocalSTAR3D is shown in Figure 6B, which only includes a small portion of each riboswitch. Another example of the circular match in riboswitches we found is between a yybP–ykoY riboswitch from *Escherichia coli* (32) and a PRPP riboswitch from *Thermoanaerobacter mathranii* (33). This alignment is shown in Supplementary Figure S4. The yybP–ykoY riboswitch binds Mn^2+^ and protects cells against Mn^2+^ toxicity by regulating a P-type ATPase (32). The PRPP riboswitch binds a different ligand, phosphoribosyl pyrophosphate (PRPP). These two riboswitches share a similar multiloop structure that contains 63 nucleotides and has an RMSD of 3.70  Å. The common structure contains their aptamer domains and can only be found by circular matching.

### Structural alignments for ribozymes

Ribozymes are RNA molecules that have catalytic functions. Circular matching may help researchers study naturally occurring ribozymes as well as design synthetic ribozymes. A circularly permutated ribozyme was designed to study the structure and function of a lariat capping ribozyme (34). We used CircularSTAR3D and LocalSTAR3D to compare the original ribozyme and its circularly permutated version. As shown in Supplementary Figure S5, LocalSTAR3D only captured the non-rotated match, while CircularSTAR3D aligned the whole ribozymes as expected.

**Figure 7.**
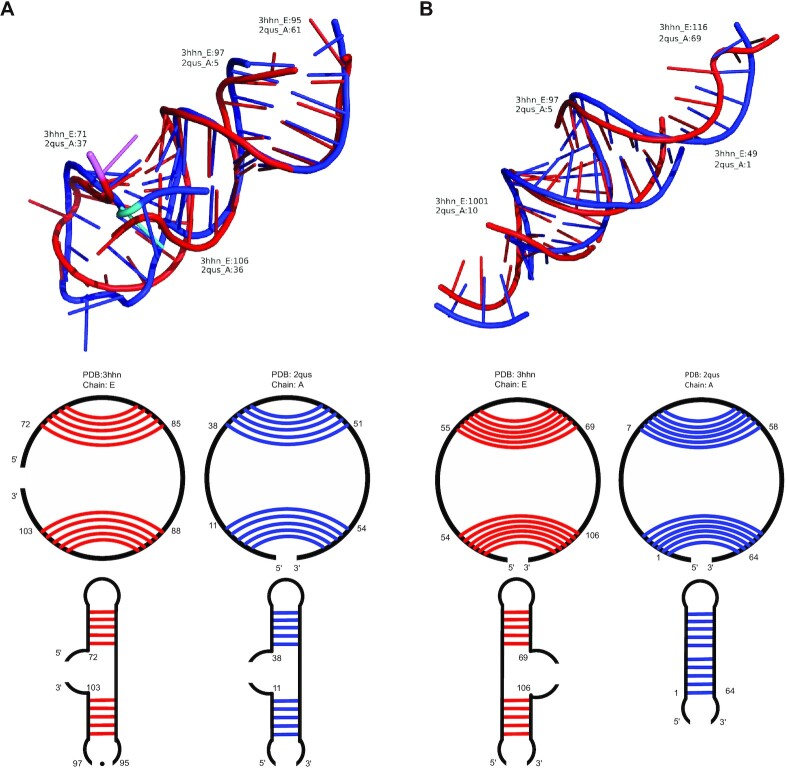
The structure alignment between a class I ligase ribozyme (PDB 3hhn, chain E) and a hammerhead ribozyme (PDB 2qus, chain A) generated by CircularSTAR3D and LocalSTAR3D. The upper part of each subfigure shows the superimposition of the aligned regions, where the red tubes represent the aligned regions in the class I ligase ribozyme and the blue tubes represent those in the hammerhead ribozyme. The lower part of each subfigure is the secondary structure of the aligned region. The secondary structures are represented by circular diagrams and cartoons, where the red and blue lines indicate the base pairs in the conserved stacks that are used as anchors in the alignment process. (**A**) The alignment generated by CircularSTAR3D. The active sites are colored violet in the class I ligase ribozyme and cyan in the hammerhead ribozyme. (**B**) The alignment generated by LocalSTAR3D.

To comprehensively identify the circular matches in ribozymes, we downloaded a set of ribozyme structures in the ‘Representative Sets of RNA 3D Structures’ list (version 3.141) (29), which contains 34 ribozyme structures. CircularSTAR3D was used to find the conserved circular matches among these ribozyme structures. CircularSTAR3D generated 27 circular alignments with positive alignment scores, out of which 16 alignments have better coverage than the best alignments of LocalSTAR3D (see Supplementary Table S2). An example of the alignments is shown in Figure 7, where the red tube is a class I ligase ribozyme (PDB 3hhn, chain E) (35) and the blue tube is a hammerhead ribozyme (PDB 2qus, chain A) (36). CircularSTAR3D found a circular match of 35 nucleotides around a rotated matched multiloop (two out of three stems are aligned). In addition, we found that the active sites of both ribozymes are at almost the same positions in the multiloop (colored violet in the class I ligase ribozyme and cyan in the hammerhead ribozyme). This similarity may suggest common features across different classes of ribozymes. LocalSTAR3D found a non-rotated match of 33 nucleotides. However, the match does not include the active sites.

## DISCUSSION

In this study, we presented a novel computational tool for circular matching, named CircularSTAR3D. It can detect the circular matches between the input RNA structures. CircularSTAR3D first identifies the rotated matched internal loops and multiloops in the input RNAs. Then it extends from all the conserved stack pairs that close these loops. This ‘two-step’ strategy is derived based on two observations. First, internal loops and multiloops can have circular matches when concatenating the loop regions in different ways. The hairpin loops only have one loop region and do not have circular alignment. Second, a pair of rotated matched loops are closed by at least one rotated matched stack pair. By integrating these two properties into the design, we developed CircularSTAR3D as a new ‘stack-based’ RNA 3D structure alignment algorithm. By using the aligned stacks as anchors, CircularSTAR3D avoids the all-to-all loop matching and enumerating different ways in which concatenation can occur. In most cases, the current implementation can find all rotated regions between two RNA 3D structures in 10 s on a modern desktop. We provide CircularSTAR3D and LocalSTAR3D in one software package so that users can generate both rotated and non-rotated matches.

Besides the alignment examples we have shown in the Results, a potential application is to integrate it into RNA clustering pipelines along with STAR3D and LocalSTAR3D. CircularSTAR3D outputs an alignment score to sort the aligned regions in a pair of RNA 3D structures. For clustering applications, we need to compare the alignments from different pairs of input RNAs, and a size-independent score should be used for that purpose, such as RMscore (11) and TM-score (12). Another direction is to integrate the long-range interaction into the local extension process. Similar to LocalSTAR3D, CircularSTAR3D considers a circular match as a collection of adjacent rotated matched stacks and loops. We can replace the sequential adjacency constraint with the spatial adjacency constraint to enable the detection of conserved components with long-range interactions, even the conserved interactions between different molecules.

## DATA AVAILABILITY

The CircularSTAR3D stack-based RNA 3D structural alignment tool is available at https://github.com/ucfcbb/CircularSTAR3D and https://doi.org/10.5281/zenodo.7719548.

## Supplementary Material

gkad222_Supplemental_Files

## References

[1] Chang Y.F. , Huang Y.L., Lu C.L. SARSA: a web tool for structural alignment of RNA using a structural alphabet. Nucleic Acids Res. 2008; 36:19–24. 10.1093/nar/gkn327 PMC2447761 18502774

[2] Wang C.W. , Chen K.T., Lu C.L. iPARTS: an improved tool of pairwise alignment of RNA tertiary structures. Nucleic Acids Res. 2010; 38:W340–W347. 20507908 10.1093/nar/gkq483 PMC2896121

[3] Yang C.H. , Shih C.T., Chen K.T., Lee P.H., Tsai P.H., Lin J.C., Yen C.Y., Lin T.Y., Lu C.L. iPARTS2: an improved tool for pairwise alignment of RNA tertiary structures, version 2. Nucleic Acids Res. 2016; 44:W328–W332. 27185896 10.1093/nar/gkw412 PMC4987943

[4] Ferre F. , Ponty Y., Lorenz W.A., Clote P. DIAL: a web server for the pairwise alignment of two RNA three-dimensional structures using nucleotide, dihedral angle and base-pairing similarities. Nucleic Acids Res. 2007; 35:W659–W668. 17567620 10.1093/nar/gkm334 PMC1933154

[5] Capriotti E. , Marti-Renom M.A. RNA structure alignment by a unit-vector approach. Bioinformatics. 2008; 24:i112–118. 18689811 10.1093/bioinformatics/btn288

[6] Capriotti E. , Marti-Renom M.A. SARA: a server for function annotation of RNA structures. Nucleic Acids Res. 2009; 37:W260–W265. 19483098 10.1093/nar/gkp433 PMC2703911

[7] Dror O. , Nussinov R., Wolfson H. ARTS: alignment of RNA tertiary structures. Bioinformatics. 2005; 21:47–53. 10.1093/bioinformatics/bti1108 16204124

[8] Ge P. , Zhang S. STAR3D: a stack-based RNA 3D structural alignment tool. Nucleic Acids Res. 2015; 43:e137. 26184875 10.1093/nar/gkv697 PMC4787758

[9] Chen X. , Khan N.S., Zhang S. LocalSTAR3D: a local stack-based RNA 3D structural alignment tool. Nucleic Acids Res. 2020; 48:e77. 32496533 10.1093/nar/gkaa453 PMC7367197

[10] Cech P. , Svozil D., Hoksza D. SETTER: web server for RNA structure comparison. Nucleic Acids Res. 2012; 40:W42–W48. 22693209 10.1093/nar/gks560 PMC3394248

[11] Zheng J. , Xie J., Hong X., Liu S. RMalign: an RNA structural alignment tool based on a novel scoring function RMscore. BMC Genom. 2019; 20:276. 10.1186/s12864-019-5631-3 PMC6454663 30961545

[12] Gong S. , Zhang C., Zhang Y. RNA-align: quick and accurate alignment of RNA 3D structures based on size-independent TM-score. Bioinformatics. 2019; 35:4459–4461. 31161212 10.1093/bioinformatics/btz282 PMC6821192

[13] Zhong C. , Tang H., Zhang S. RNAMotifScan: automatic identification of RNA structural motifs using secondary structural alignment. Nucleic Acids Res. 2010; 38:e176. 20696653 10.1093/nar/gkq672 PMC2952876

[14] Zhong C. , Zhang S. Clustering RNA structural motifs in ribosomal RNAs using secondary structural alignment. Nucleic Acids Res. 2012; 40:1307–1317. 21976732 10.1093/nar/gkr804 PMC3273805

[15] Zhong C. , Zhang S. RNAMotifScanX: a graph alignment approach for RNA structural motif identification. RNA. 2015; 21:333–346. 10.1261/rna.044891.114 PMC4338331 25595715

[16] Ge P. , Islam S., Zhong C., Zhang S. De novo discovery of structural motifs in RNA 3D structures through clustering. Nucleic Acids Res. 2018; 46:4783–4793. 29534235 10.1093/nar/gky139 PMC5961109

[17] Roth A. , Weinberg Z., Vanderschuren K., Murdock M.H., Breaker R.R. Natural circularly permuted group II introns in bacteria produce RNA circles. iScience. 2021; 24:103431. 34901790 10.1016/j.isci.2021.103431 PMC8637638

[18] Mustafina K. , Nomura Y., Rotrattanadumrong R., Yokobayashi Y. Circularly-permuted pistol ribozyme: a synthetic ribozyme scaffold for mammalian riboswitches. ACS Synth. Biol. 2021; 10:2040–2048. 34374523 10.1021/acssynbio.1c00213

[19] Weinberg C.E. , Olzog V.J., Eckert I., Weinberg Z. Identification of over 200-fold more hairpin ribozymes than previously known in diverse circular RNAs. Nucleic Acids Res. 2021; 49:6375–6388. 34096583 10.1093/nar/gkab454 PMC8216279

[20] Lu X.J. , Bussemaker H.J., Olson W.K. DSSR: an integrated software tool for dissecting the spatial structure of RNA. Nucleic Acids Res. 2015; 43:e142. 26184874 10.1093/nar/gkv716 PMC4666379

[21] Bron C. , Kerbosch J. Algorithm 457: finding all cliques of an undirected graph. Commun. ACM. 1973; 16:575–577.

[22] Lescoute A. , Leontis N.B., Massire C., Westhof E. Recurrent structural RNA motifs, isostericity matrices and sequence alignments. Nucleic Acids Res. 2005; 33:2395–2409. 15860776 10.1093/nar/gki535 PMC1087784

[23] Klein D.J. , Moore P.B., Steitz T.A. The roles of ribosomal proteins in the structure assembly, and evolution of the large ribosomal subunit. J. Mol. Biol. 2004; 340:141–177. 15184028 10.1016/j.jmb.2004.03.076

[24] Huang L. , Lilley D.M. The molecular recognition of kink-turn structure by the L7Ae class of proteins. RNA. 2013; 19:1703–1710. 24149842 10.1261/rna.041517.113 PMC3884654

[25] Ren A. , Kosutic M., Rajashankar K.R., Frener M., Santner T., Westhof E., Micura R., Patel D.J. In-line alignment and Mg^2+^ coordination at the cleavage site of the env22 twister ribozyme. Nat. Commun. 2014; 5:5534. 25410397 10.1038/ncomms6534 PMC4373348

[26] Roth A. , Weinberg Z., Chen A.G., Kim P.B., Ames T.D., Breaker R.R. A widespread self-cleaving ribozyme class is revealed by bioinformatics. Nat. Chem. Biol. 2014; 10:56–60. 24240507 10.1038/nchembio.1386 PMC3867598

[27] Roth A. , Breaker R.R. The structural and functional diversity of metabolite-binding riboswitches. Annu. Rev. Biochem. 2009; 78:305–334. 19298181 10.1146/annurev.biochem.78.070507.135656 PMC5325118

[28] Breaker R.R. Riboswitches and the RNA world. Cold Spring Harb. Perspect. Biol. 2012; 4:a003566. 21106649 10.1101/cshperspect.a003566 PMC3281570

[29] Leontis N. , Zirbel C. Leontis N. , Westhof E. Nonredundant 3D structure datasets for RNA knowledge extraction and benchmarking. RNA 3D Structure Analysis and Prediction. 2012; Berlin Heidelberg Springer 281–298.

[30] Johnson J.E. , Reyes F.E., Polaski J.T., Batey R.T. B12 cofactors directly stabilize an mRNA regulatory switch. Nature. 2012; 492:133–137. 23064232 10.1038/nature11607 PMC3518761

[31] Serganov A. , Huang L., Patel D.J. Coenzyme recognition and gene regulation by a flavin mononucleotide riboswitch. Nature. 2009; 458:233–237. 19169240 10.1038/nature07642 PMC3726715

[32] Price I.R. , Gaballa A., Ding F., Helmann J.D., Ke A. Mn^2+^-sensing mechanisms of yybP–ykoY orphan riboswitches. Mol. Cell. 2015; 57:1110–1123. 25794619 10.1016/j.molcel.2015.02.016 PMC4703321

[33] Knappenberger A.J. , Reiss C.W., Strobel S.A. Structures of two aptamers with differing ligand specificity reveal ruggedness in the functional landscape of RNA. Elife. 2018; 7:e36381. 29877798 10.7554/eLife.36381 PMC6031431

[34] Meyer M. , Nielsen H., Oliéric V., Roblin P., Johansen S. D., Westhof E., Masquida B. Speciation of a group I intron into a lariat capping ribozyme. Proc. Natl Acad. Sci. USA. 2014; 111:7659–7664. 24821772 10.1073/pnas.1322248111 PMC4040574

[35] Shechner D.M. , Grant R.A., Bagby S.C., Koldobskaya Y., Piccirilli J.A., Bartel D.P. Crystal structure of the catalytic core of an RNA-polymerase ribozyme. Science. 2009; 326:1271–1275. 19965478 10.1126/science.1174676 PMC3978776

[36] Chi Y.I. , Martick M., Lares M., Kim R., Scott W.G., Kim S.H. Capturing hammerhead ribozyme structures in action by modulating general base catalysis. PLoS Biol. 2008; 6:e234. 18834200 10.1371/journal.pbio.0060234 PMC2553840

