# Supplementary material for: CircularSTAR3D: a stack-based RNA 3D structural alignment tool for circular matching
Source: Nucleic Acids Res. 2023 Mar 29;51(9):e53. doi: 10.1093/nar/gkad222 (PMC10201423; doi:10.1093/nar/gkad222)
Supplement: gkad222_Supplemental_Files [file gkad222_supplemental_files.zip › nar_CircularSTAR3D_supplementary.pdf]

Supplementary Data for ‘CircularSTAR3D: a stack-based RNA 3D structural alignment tool for circular matching’

Xiaoli Chen and Shaojie Zhang *

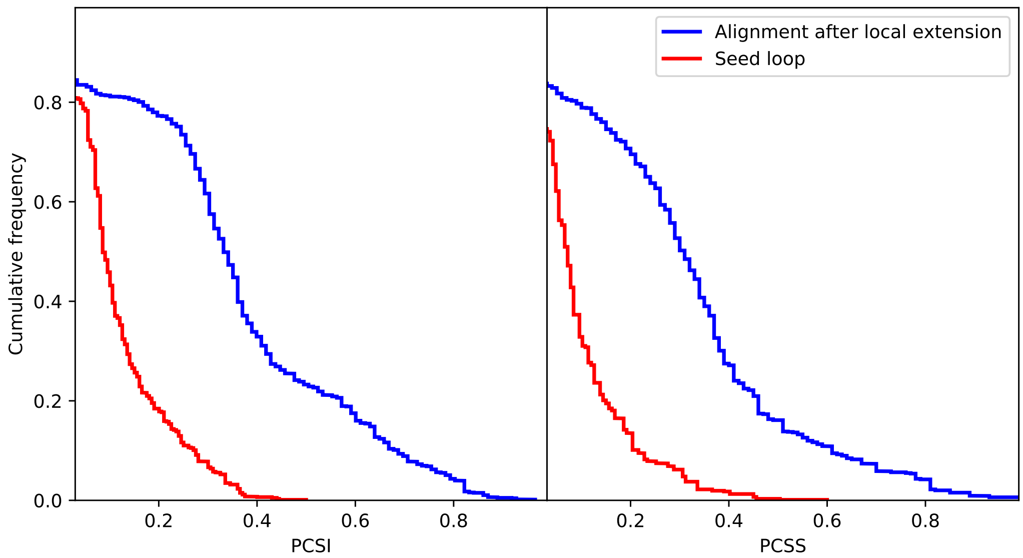

Figure S1: The cumulative frequencies of the PCSI and the PCSS values in CircularSTAR3D’s second-best alignments. The blue curves represent the PCSI and the PCSS values in the alignments after the local extension. The red curves represent the PCSI and the PCSS values in the seed loops.

*To whom correspondence should be addressed. Tel: +1 407 823 6095; Fax: +1 407 823 5835; Email: shzhang@cs.ucf.edu

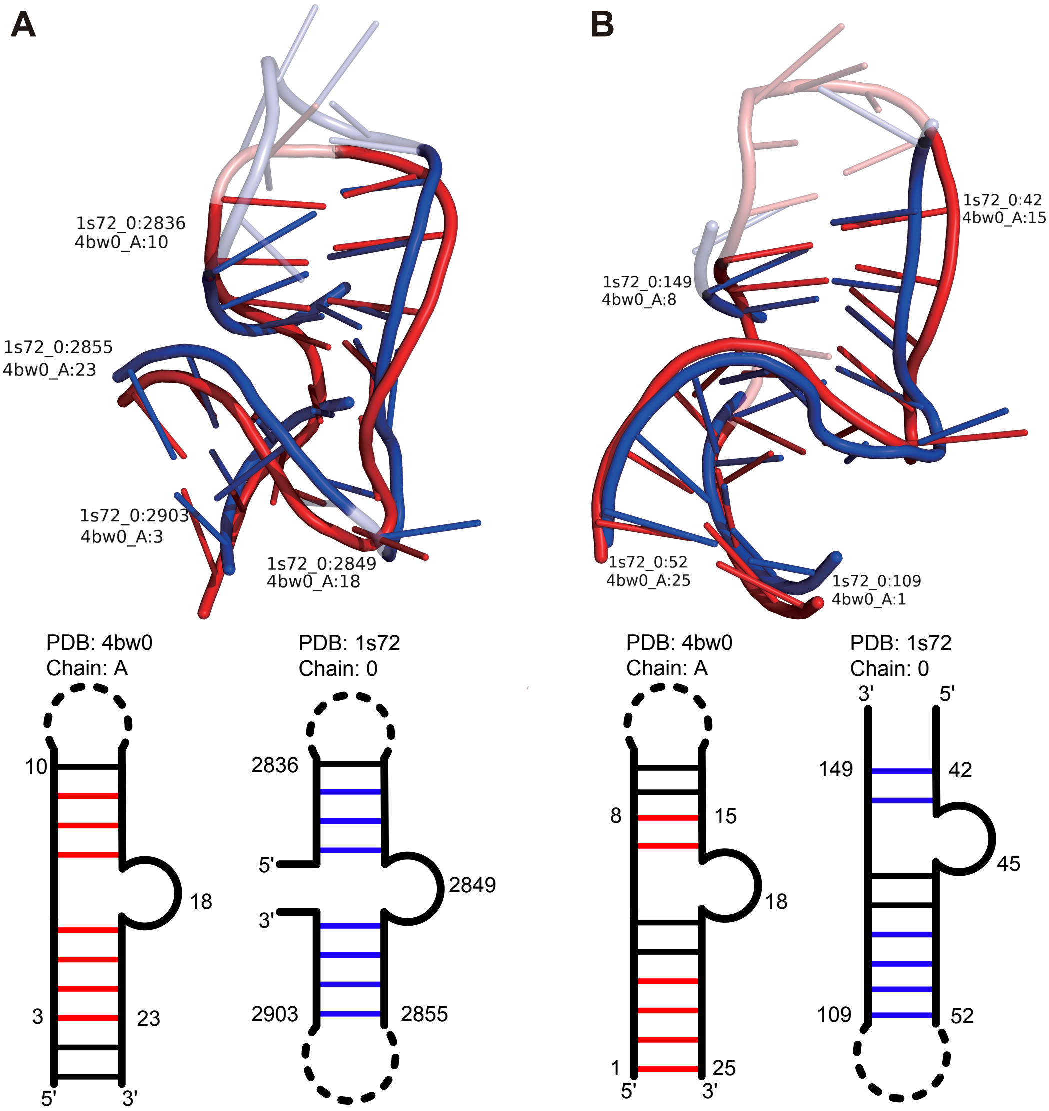

Figure S2: The circular matching instances for a kink-turn motif on *H. marismortui* 23S rRNA (PDB 1s72, chain 0). The upper part of each subfigure shows the super-imposition of the aligned regions, where the red tubes represent the kink-turn query structure and the blue tubes represent the rRNA. The insertions and deletions in the alignment are shown in half-transparent tubes. The lower part of each subfigure shows the secondary structures of the aligned regions, in which the kink-turn query is on the left and the rRNA is on the right. In the secondary structures, the red lines indicate the base pairs in the conserved stacks that are used as anchors in the alignment process, the solid lines show the aligned regions, and the dashed lines are the rest of the regions for completeness. (**A**) The first kink-turn motif circular match instance. (**B**) The second kink-turn motif circular match instance.

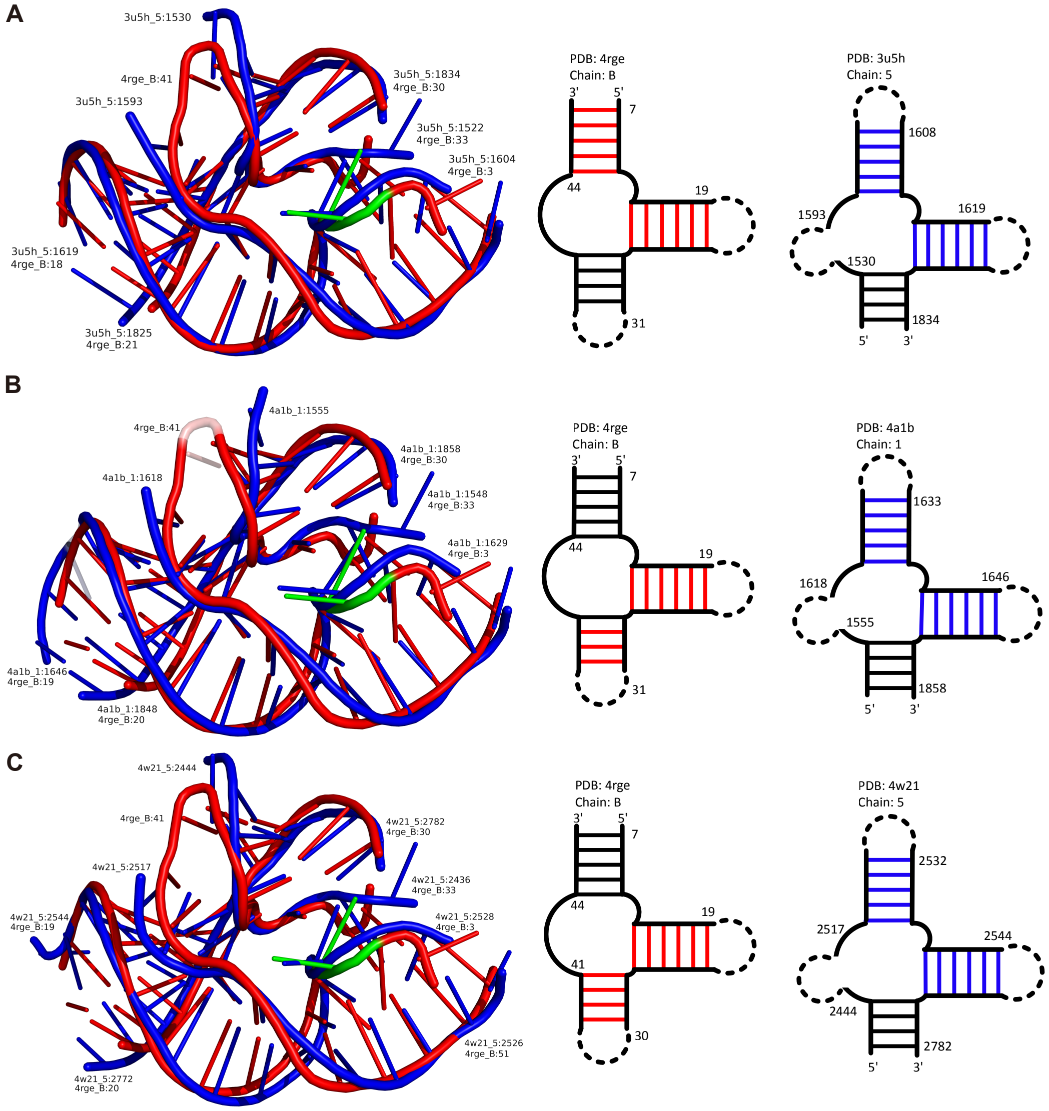

Figure S3: Circular matching instances of a multi-loop motif. The left part of each subfigure shows the super-imposition of the aligned region, where the red tubes represent the aligned regions in the twister ribozyme and the blue tubes represent that in rRNAs. The insertions and deletions in the alignment are shown in half-transparent tubes. The self-cleavage sites in the twister ribozyme are highlighted by green color. The right part of each subfigure shows the secondary structures of the aligned regions, in which the twister ribozyme is on the left and the rRNA is on the right. In the secondary structures, the red lines and the blue lines indicate the base pairs in the conserved stacks that are used as anchors in the alignment process, the solid lines show the aligned regions, and the dashed lines are the rest of the regions for completeness. (**A**) The motif instance in *S. cerevisiae* 25S rRNA (PDB 3u5h, chain 5). (**B**) The motif instance in *T. thermophila* 26S rRNA (PDB 4a1b, chain 1). (**C**) The motif instance in *S. scrofa* 28S rRNA (PDB 4w21, chain 5).

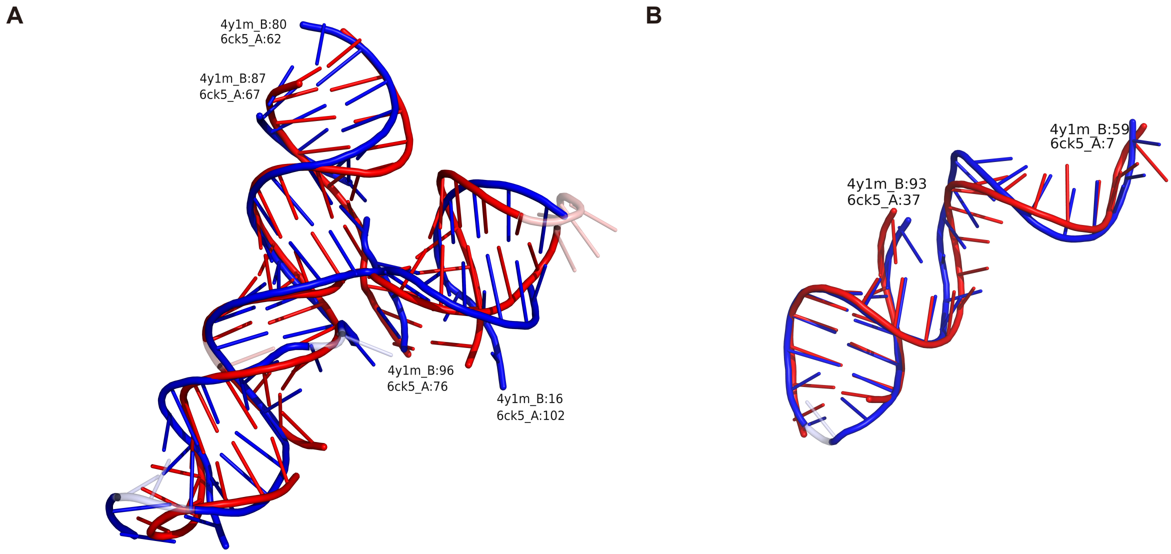

Figure S4: The structure alignment between a yybP-ykoY riboswitch (PDB 4y1m, chain B) and a PRPP riboswitch (PDB 6ck5, chain A) generated by CircularSTAR3D and LocalSTAR3D. The red tubes represent the aligned regions in the yybP-ykoY riboswitch and the blue tubes represent those in the PRPP riboswitch. The insertions and deletions in the alignment are shown in half-transparent tubes. (**A**) The alignment generated by CircularSTAR3D. (**B**) The alignment generated by LocalSTAR3D.

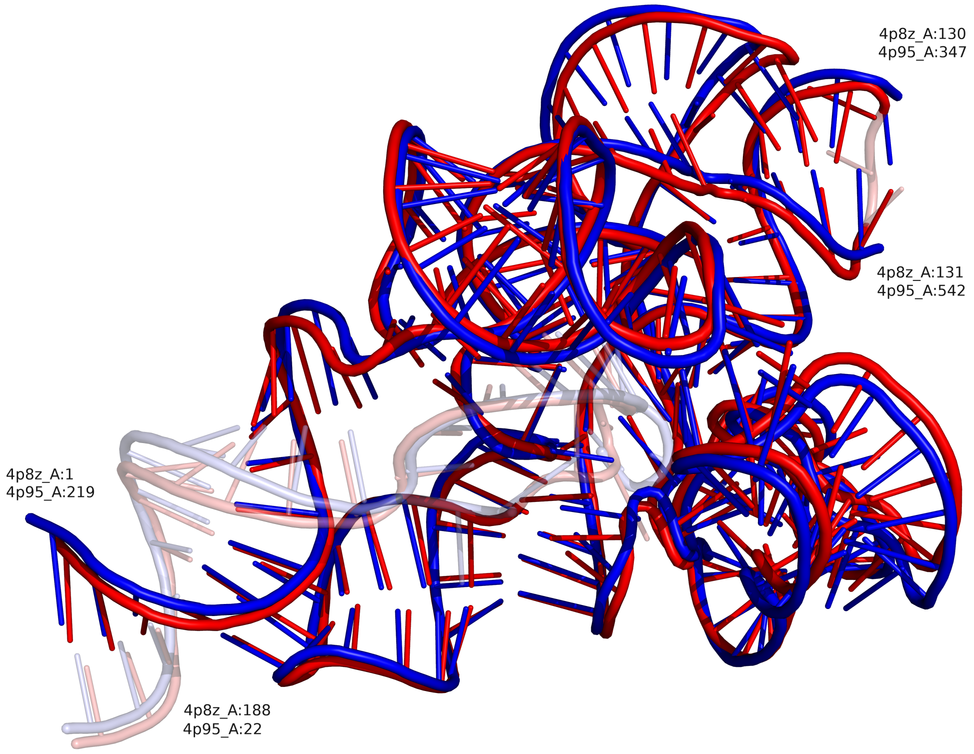

Figure S5: The structure alignment between a lariat capping ribozyme (PDB 4p8z, chain A) and its circularly permutated version (PDB 4p95, chain A). The red tubes represent the lariat capping ribozyme and the blue tubes represent the circularly permutated version. The aligned region generated by LocalSTAR3D are shown in opaque tubes. The additional aligned regions generated by CircularSTAR3D are shown in half-transparent tubes.
